# Are Religious Grandparents More Involved Grandparents Because Their Values Are Less Individualistic?

**DOI:** 10.1093/geroni/igaf122.955

**Published:** 2025-12-31

**Authors:** Martin Lakomy, Merril Silverstein, Tianqi Zhou, Jeewon Oh

**Affiliations:** Mendel University in Brno, Brno, Czech Republic; Syracuse University, Syracuse, New York, United States; Syracuse University, Syracuse, New York, United States; Syracuse University, Syracuse, New York, United States

## Abstract

This investigation examined whether middle-aged and older adults who hold stronger religious values are more likely to provide instrumental and emotional support to their grandchildren, and whether this relationship is mediated by lesser importance attributed to self-interested or individualistic values. We used data from Waves 4-10 of the Longitudinal Study of Generations to examine the link between religious values, individualistic values, and support provided using mixed models with mediating effects. The operational sample consisted of 3,568 observations from 1,340 respondents nested within 305 families. Values were measured with eight items from the Rokeach Values Survey a scale ranking various intrinsic goals, ordered by their degree of importance. The scale’s dimension of individualism-collectivism is used to assess the importance of self-aggrandizing goals associated with individualism—such as achievement and skills—as opposed to social goals associated with collectivism—such as friendship and community. Religious intensity—a measure of the religious self-concept—is considered as a lagged indicator taken prior to the respondent becoming a grandparent. While we expect individualistic values to weaken with aging, the degree of the connection between religion and those values may be manifest in greater support that directly benefits grandchildren and indirectly their adult children.

